# First report of natural *Wolbachia* infection in the malaria mosquito *Anopheles arabiensis* in Tanzania

**DOI:** 10.1186/s13071-018-3249-y

**Published:** 2018-12-13

**Authors:** Francesco Baldini, Justine Rougé, Katharina Kreppel, Gustave Mkandawile, Salum Abdallah Mapua, Maggy Sikulu-Lord, Heather M. Ferguson, Nicodem Govella, Fredros O. Okumu

**Affiliations:** 1 Institute of Biodiversity Animal Health and Comparative Medicine, University of Glasgow, Glasgow, G12 8QQ UK; 2 Environmental Health & Ecological Sciences Department, Ifakara Health Institute, Off Mlabani Passage, PO Box 53, Ifakara, Tanzania; 3 The School of Public Health, The University of Queensland, Sydney, Queensland Australia; 4 School of Public Health, Faculty of Health Sciences, University of the Witwatersrand, Johannesburg, South Africa

**Keywords:** *Wolbachia*, *Anopheles*, Malaria parasite, Endosymbiont, Pathogen interference, Maternal transmission, Vector control, Tanzania

## Abstract

**Background:**

Natural infections of the endosymbiont bacteria *Wolbachia* have recently been discovered in populations of the malaria mosquito *Anopheles gambiae* (*s.l*.) in Burkina Faso and Mali, West Africa. This *Anopheles* specific strain *w*Anga limits the malaria parasite *Plasmodium falciparum* infections in the mosquito, thus it offers novel opportunities for malaria control.

**Results:**

We investigated *Wolbachia* presence in *Anopheles arabiensis* and *Anopheles funestus*, which are the two main malaria vectors in the Kilombero Valley, a malaria endemic region in south-eastern Tanzania. We found 3.1% (*n* = 65) and 7.5% (*n* = 147) *w*Anga infection prevalence in *An. arabiensis* in mosquitoes collected in 2014 and 2016, respectively, while no infection was detected in *An. funestus* (*n* = 41). Phylogenetic analysis suggests that at least two distinct strains of *w*Anga were detected, both belonging to *Wolbachia* supergroup A and B.

**Conclusions:**

To our knowledge, this is the first confirmation of natural *Wolbachia* in malaria vectors in Tanzania, which opens novel questions on the ecological and genetic basis of its persistence and pathogen transmission in the vector hosts. Understanding the basis of interactions between *Wolbachia*, *Anopheles* mosquitoes and malaria parasites is crucial for investigation of its potential application as a biocontrol strategy to reduce malaria transmission, and assessment of how natural *w*Anga infections influence pathogen transmission in different ecological settings.

**Electronic supplementary material:**

The online version of this article (10.1186/s13071-018-3249-y) contains supplementary material, which is available to authorized users.

## Background

The maternally inherited endosymbiont bacteria *Wolbachia* infects an estimated 40 to 66% of all insect species worldwide [1, 2]. To ensure its transmission and spread in naive insect populations, *Wolbachia* has, in some species, been found to alter reproduction of the insect host to favour female progeny. For example, it induces production of only female progeny, parthenogenesis and cytoplasmic incompatibility (CI) (i.e. the embryonic death of offspring) from *Wolbachia*-infected males and uninfected females [3]. *Wolbachia* has been proposed as a biocontrol tool against vector-borne diseases because it can reduce the pathogens developing within insect vectors. For example, *Aedes aegypti* mosquitoes that were laboratory infected with *Wolbachia* are unable to sustain infections with dengue (DENV) [4] and Zika (ZIKV) viruses [5]. By exploiting the CI phenotype of *Wolbachia*, endosymbiont infected *Ae. aegypti* have since been introduced and subsequently spread into natural mosquito populations with the aim of reducing dengue and Zika transmission [6, 7].

While *Ae. aegypti* mosquitoes are naturally uninfected with *Wolbachia* [8], other mosquito species carry natural infections of this endosymbiont, for example *Culex pipiens* [9] and *Aedes albopictus* [10]. Recently, the major African malaria vectors of the *Anopheles gambiae* (*s.l*.) complex [including *An. gambiae* (*s.s*.), *An. coluzzii* and *An. arabiensis*] were found to be infected in Burkina Faso [11–13] and Mali [14]. Additional investigations detected *Wolbachi*a in other malaria vectors also in Central and East Africa [15, 16]. These findings suggest that in addition to artificially introduced *Wolbachia* strains in the laboratory [17], natural infections in *Anopheles* mosquitoes should be exploited to identify any opportunities for malaria control. Indeed, negative associations between *w*Anga (the *Anopheles*-specific *Wolbachia* strain/s) and the human malaria parasite *Plasmodium falciparum* were found in *An. gambiae* (*s.l*). [13, 14]. Additionally, *An. coluzzii* with natural *w*Anga infections were at least two times less likely to harbour the malaria parasite once experimentally infected with *P. falciparum*, suggesting a protective effect of the endosymbiont against this pathogen in the mosquito [14]. These early findings raise prospects for the future application of *w*Anga for malaria control. However, such a strategy will require extensive knowledge of the biology of natural *w*Anga infections in malaria vectors, including the genetic and ecological basis of the induced phenotypes and the mechanisms of parasite interference.

One key aspect of *w*Anga biology that needs to be elucidated is its mechanism of persistence and transmission in the mosquito populations. Maternal transmission seems to be incomplete [11], suggesting that this strain is associated with a strong fitness benefit to the female progeny, or that additional factors may be required to ensure successful transgenerational transmission and survival. Nevertheless, laboratory investigations using *w*Anga infected *Anopheles* mosquitoes showed that the endosymbiont does not induce CI [13, 14] or distortion of sex ratio [13]. Further work is required to understand if the lack of CI would also occur under natural settings. One apparent fitness advantage of *w*Anga is the observed accelerated oviposition timing, which could increase the number of gonotrophic cycles and therefore the total number of progeny; nevertheless, this increased oviposition rate might be associated with a decrease in lifespan [18], thus the actual fitness benefit of this induced phenotype is still not resolved.

The identification of natural infections under different ecological settings and in different vector species is crucial to understand the potential impact of this endosymbiont on disease transmission dynamics, and how it could be exploited for vector control. As *Wolbachia*-induced phenotypes depend on the co-evolutionary history of the host and endosymbiont [19], exploiting the natural *Wolbachia*-induced parasite interference in *Anopheles* might result in a more sustainable biological control tool than using artificial infections. Consequently, it is paramount to detect and characterise natural *Wolbachia* infections in *Anopheles* populations. Here, we investigated the presence of *Wolbachia* in *An. arabiensis* and *An. funestus* in the Kilombero Valley, south-eastern Tanzania, where these two species are the dominant malaria vectors [20, 21].

## Methods

### Mosquito collection and *Wolbachia* detection

**Fig. 1:**
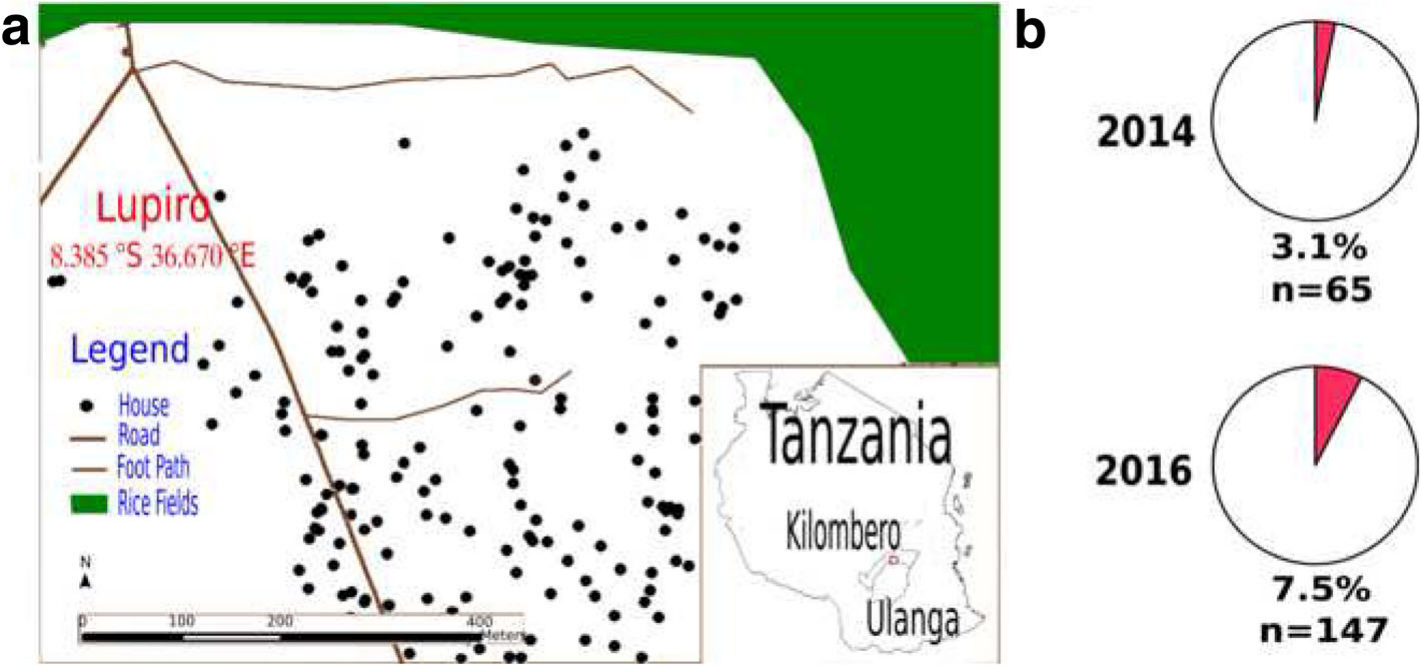
**a** Map showing Lupiro village (8°22'59"S, 36°40'00"E) in Ulanga district, south-eastern Tanzania, from where the *Anopheles* specimens were obtained (Courtesy of Alex J. Limwagu, Ifakara Health Institute). **b** The prevalence of *Wolbachia* in *An. arabiensis* in Lupiro village, in 2014 and 2016, is indicated

Collections were performed in Lupiro village (8°22'59"S, 36°40'00"E) in Ulanga district, south-eastern Tanzania (Fig. 1a), in November 2014 and in July 2016, during rainy and dry seasons, respectively. Major *Anopheles* in the area include the *An. funestus* (*s.l.*) group [including *An. funestus* (*s.s*.) Giles, *An. leesoni* and *An. rivulorum*] and the *An. gambiae* (*s.l.*) complex (consisting primarily of *An. arabiensis*), *An. coustani*, *An. pharoensis*, *An. squamosus*, *An. ziemanni* and *An. wellcomei*. Of these the main malaria vectors include *An. funestus* (*s.s*.) and *An. arabiensis* with minor contributions from *An. rivulorum*. Overall the entomological inoculation rate (EIR) was last estimated at 4.2 and 11.7 infectious bites/person/year by *An. arabiensis* and *An. funestus*, respectively. There are also culicine species, mainly *Mansonia*, *Aedes* and *Culex* mosquito species [20, 22]. Adult female *Anopheles* mosquitoes were collected either inside houses with CDC light traps (Prevention, C.f.D.C.a., Model 512, John Hock, Gainesville, FL, USA) or outdoor with backpack aspirators (Prevention, C.f.D.C.a., Model 1412, John Hock). Mosquitoes were sampled from collections from 10 houses. *An. gambiae* (*s.l.*) complex and *An. funestus* (*s.l.*) group were morphologically identified and DNA extracted from individual whole fresh mosquitoes using a DNeasy kit (Qiagen, Manchester, UK) and eluted in 50 μl of water. Forty to 120 ng of DNA was used to amplify the *Wolbachia*-specific *16S* rDNA region using an established nested PCR approach for natural *w*Anga infections in *An. gambiae* (*s.l*.) [13]. All 13 amplified 412-bp fragments were confirmed to correspond to *Wolbachia* by Sanger sequencing (Eurofins Genomics, Ebersberg, Germany) (GenBank accession numbers MH596693-MH596703). PCR was used to identify species in *An. gambiae* (*s.l.*) complex [23] and *An. funestus* (*s.l.*) group [24].

### Phylogenetic analysis

*Wolbachia 16S* rRNA sequences were aligned using Clustal Omega [25]. Other *Wolbachia* sequences comprising members of the supergroups A (*w*Mel AE017196.1, *w*Ri CP001391.1, *w*Ha CP003884.1), B (*w*Pip AM999887.1, *w*AlbB KX155506.1, *w*No CP003883.1), C (*w*Oo AJ010276.1), D (*w*Bm AE017321.1) and *w*Anga (*w*Anga_BF: KP089991 in *An. coluzzii* [12], KJ728740.1 and KJ728755.1 in *An. coluzzii* [11], KJ728754.1 in *An. gambiae* [11], *w*Anga_Mali: MF944114.1 in *An. gambiae* [14], *w*Anga_TZ: MH596693, MH596696, MH596697, MH596703 in *An. arabiensis*) sequences were included (Additional file 1: Figure S1).

The sequences of the endosymbionts *Rickettsia japonica* (CP032049.1), *Ehrlichia chaffeensis* (NR_074500.2) and *Anaplasma phagocytophilum* (KY114936.1) were included as non-*Wolbachia* reference outgroups. The general time reversible (GTR+G) model was used to calculate sequence divergences [26]. A maximum likelihood tree using 1000 bootstrap replicates of GTR+G distances was created to provide a graphic representation of the patterning of divergences among the sequences obtained from the samples.

## Results

All 212 *An. gambiae* (*s.l.*) females collected in 2014 and 2016 were identified as *An. arabiensis* by PCR. *Wolbachia*-specific *16S* rRNA nested PCR followed by sequencing (GenBank accession numbers MH596693-MH596703) identified *Wolbachia* in 3.1% (2/65) and 7.5% (11/147) of the samples collected in 2014 and 2016, respectively (Fig. 1). All 41 *An. funestus* (*s.l.*) females collected in 2014 were identified as *An. funestus *(*s.s*.) and *Wolbachia* infection was not detected. The 2016 analysis did not include any *An. funestus* mosquitoes.

**Fig. 2:**
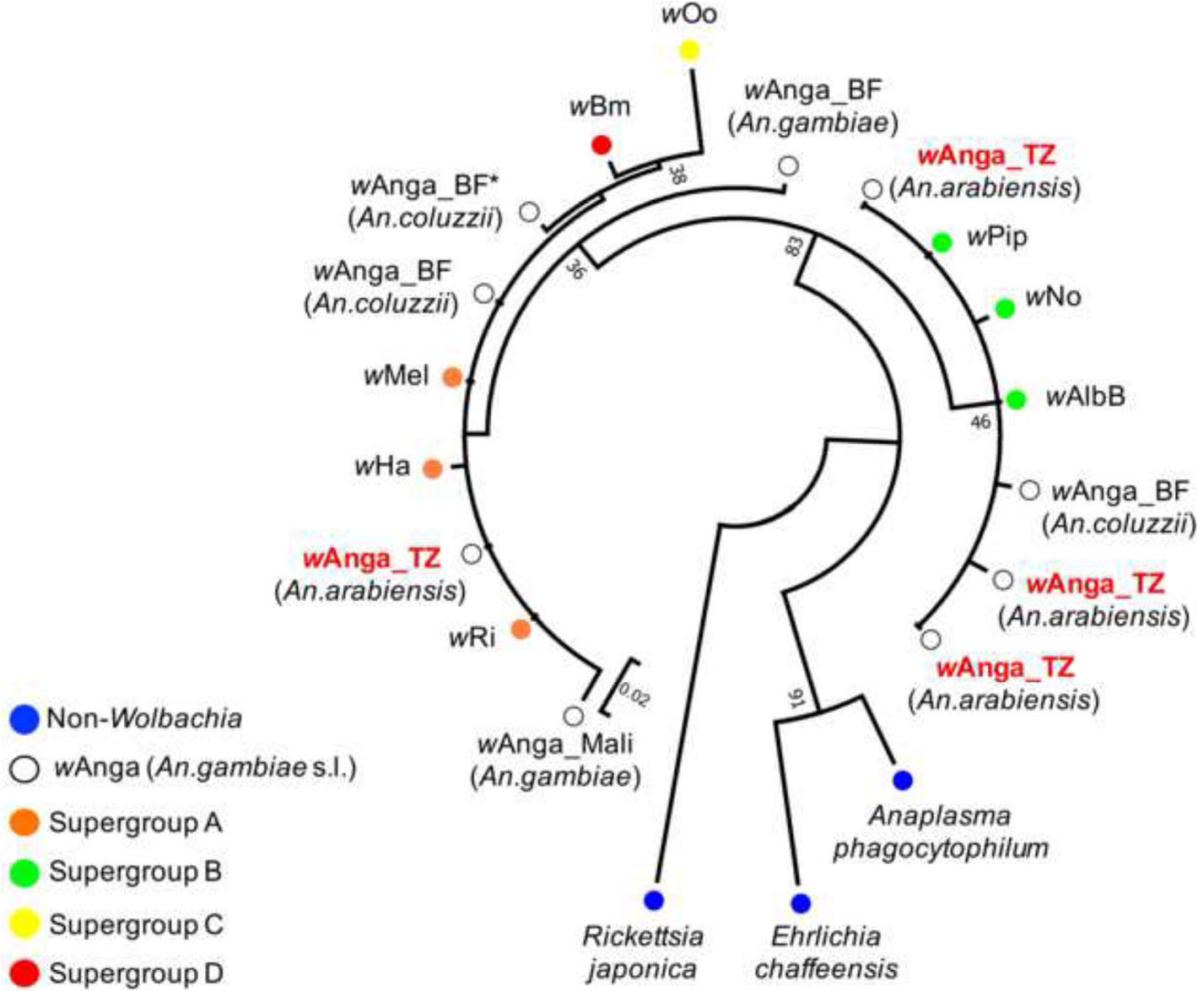
Phylogenetic analysis of the *Wolbachia*-specific *16S* rRNA conserved region. The sequences identified in this study in *An. arabiensis* in Tanzania (*w*Anga_TZ) (highlighted in red) clustered with *Wolbachia* strains from the supergroup A or B. Sequences from other *w*Anga from *An. gambiae* (*s.l.*) in Burkina Faso [11] (the asterisk indicates a sequence from Buck et al. [12]) and Mali [14] were also included. Other non-*Wolbachia* proteobacteria (*R. japonica*, *E. chaffeensis* and *A. phagocytophilum*) were also included, and the *R. japonica* sequence was used as the reference outgroup

To determine the genetic variation and diversity of the identified *Wolbachia* strain/s, which we will refer to as *w*Anga_TZ, we conducted phylogenetic analyses on 4 samples based on the conserved *16S* rRNA region amplified and sequenced. For comparison, we included other *w*Anga sequences identified in *An. gambiae* and *An. coluzzii* in Burkina Faso (*w*Anga_BF) [11, 12] and Mali (*w*Anga_Mali) [14], and sequences from arthropod-specific (subgroups A: *w*Mel, *w*Ha, *w*Ri; and B: *w*Pip, *w*AlbB, *w*No) and nematode-specific (subgroups C: *w*Oo; and D: *w*Bm) *Wolbachia* (Additional file 1: Figure S1). Most of the *w*Anga_TZ sequences (3 out of 4) clustered with supergroup B, and only one with supergroup A. Conversely, *w*Anga_Mali and most of *w*Anga_BF clustered with supergroup A and only one *w*Anga_BF from *An. coluzzii* belonged to supergroup B (Fig. 2). This phylogenetic analysis suggests that *w*Anga belongs to the supergroups A or B and exhibits a relatively high genetic diversity which is widespread in both West and East Africa.

## Discussion

Here we detected natural *Wolbachia* infections in *An. arabiensis* population in south-eastern Tanzania. To our knowledge, this is the first identification of this endosymbiont in natural populations of malaria vectors in Tanzania and highlights need for further investigation of its distribution and importance in the region. Until recently, *Wolbachia* had not been detected in natural populations of *Anopheles* mosquitoes [27–30], the vectors of human malaria. This lack of identification was probably due to a general low infection prevalence and *Wolbachia* density within species of this mosquito genus, which could have prevented the detection in the low sample sizes tested by single PCR. Both the nested PCR approach [13], which was used here, and quantitative PCR [14] increase sensitivity and are therefore more appropriate for the detection of low prevalence and low density endosymbiont loads typical of *w*Anga. Here, *Wolbachia* infection prevalence in *An. arabiensis* (3.1–7.5%, Fig. 1) was lower than *w*Anga in West Africa, where up to 33% of *An. arabiensis* were infected in the Soumousso village in Burkina Faso [13]. Furthermore, other species of the *An. gambiae* (*s.l.*) complex in West Africa (Burkina Faso and Mali) show higher infection prevalence ranging between 19–78% [13, 14]. These results suggest that natural *Wolbachia* infections are widespread in species of the *An. gambiae* (*s.l.*) complex in Africa, although their prevalence is highly variable.

We did not detect *Wolbachia* in any of the 41 *An. funestus* specimens examined. However, given the low prevalence rates observed in *An. funestus* in another study (5%) [15], the failure to detect *Wolbachia* in the *An. funestus* mosquitoes in the present study should not be interpreted as absence of the endosymbiont in this species. Larger sample sizes of *An. funestus* will need to be tested before any such conclusion can be made. However, one possible hypothesis worth investigating is that the potential absence of *w*Anga in *An. funestus* and its presence in *An. arabiensis*, coupled with proven interference of *P. falciparum* infections in some mosquitoes by *w*Anga, may be associated with the differential importance of these two species in the malaria transmission dynamics in East Africa. Indeed, although it occurs in far lower densities than *An. arabiensis*, *An. funestus* now mediates more than 80% of malaria transmission in the Kilombero Valley [20]. Future studies should thus investigate interactions and differential effects on vector competence.

As *w*Anga might have an effect on mosquito vectorial capacity [13, 14], it is crucial to understand the ecological and genetic determinants of *w*Anga infection dynamics. For example, laboratory investigations showed that in *An. stephensi* maternal transmission of an artificially introduced *Wolbachia* strain (*w*AlbB) is prevented by some components of the mosquito microbiota [31]. Furthermore, in *Drosophila*, environmental factors such as temperature and diet influence *Wolbachia* density [32, 33], potentially affecting infection dynamics by influencing maternal transmission efficiency [34] and reproduction manipulation [35]. It is therefore possible that environmental variation including microbiome composition can impede or sustain *Wolbachia* transmission in *Anopheles*. Additionally, variation in the genetic background and physiology of mosquito populations might affect *Wolbachia* persistence; indeed, in the mosquito *Culex pipiens*, the physiological costs associated with insecticide resistance results in decreased ability to control *Wolbachia* infection and consequently increased endosymbiont density [36, 37]. Thus, the widespread insecticide resistance occurring in malaria vectors in Africa [38] could also be responsible for the spread of *Wolbachia* into *Anopheles* populations, possibly reducing malaria transmission. Additional investigations under different ecological settings and mosquito host genetic backgrounds (including presence and absence of different insecticide resistance mechanisms) are therefore required to understand which factors are affecting *w*Anga infection dynamics and ultimately the vectorial capacity of its malaria vector hosts.

Understanding the mechanisms and genetic basis of *w*Anga induced parasite interference is also imperative. Elucidating *w*Anga genetic variation and association with parasite infection could be a first step to unravel the molecular bases of this phenotype and any associated drivers of parasite interference. Here, phylogenetic analysis of the conserved *16S* rRNA region showed that at least two strains infect *An. arabiensis* in Tanzania, and that both strains belong to either supergroup A or B (Fig. 2). Multilocus sequence typing (MLST) and/or whole genome sequencing of different *w*Anga isolates will be required to fully characterize the genetic diversity of the circulating strains. Genetic characterization is crucial, as different strains can have opposite effects on malaria parasites, as observed in *Anopheles* species that were artificially infected with different *Wolbachia* strains and experimentally challenged with *Plasmodium* in the laboratory [39] (Table 1). Indeed, pathogen inhibition may not be a consistent consequence of *Wolbachia* infection. For example, natural *Wolbachia* infections can increase the susceptibility of *Aedes* and *Culex* mosquitoes and *Simulium* blackflies to avian malaria parasites [40–42]. Therefore, it will be crucial to assess the impact of *w*Anga on malaria infections and vectorial capacity under natural, ecologically variable conditions.

**Table 1:** *Wolbachia* dependent phenotypes in *Anopheles.* The phenotypes of different *Wolbachia* strains infecting *Anopheles* species are summarized. ↑, ↓, = indicate increased, decreased or stable associations or influence on the trait/phenotype, respectively. CI indicates cytoplasmic incompatibity. One asterisks refers to induced maternal transmission by microbiome suppression [31], two asterisks to a temperature dependent phenotype [43], three asterisks refer to the present study

*Wolbachia* strain	*Anopheles* species	Type of infection	Maternal transmission	CI	*Plasmodium* infection	Other phenotypes	Reference
*w*AlbB	*An. stephensi*	Artificial	Yes	Yes	*P. falciparum*: ↓oocysts; ↓ sporozoites	↑ immune response	[17, 44]
					*P. berghei*: ↓ oocysts; ↓ sporozoites		
*w*AlbB	*An. gambiae*	Artificial	No/Yes^*^	No	*P. falciparum*: ↓ oocysts	↑/↓immune response	[31, 45, 46]
					*P. berghei*: ↑ oocysts		
*w*AlbB	*An. stephensi*	Artificial	No/Yes^*^	No	*P. yoelii*: ↑/↓ oocysts^**^; ↑/↓ sporozoites^**^	↑ immune response^**^	[31]
*w*MelPop	*An. gambiae*	Artificial	No	No	*P. falciparum*: ↓ oocysts	↑ immune response	[45–47]
					*P. berghei*: ↓/= oocysts	↓ survival	
*w*Anga_BF	*An. gambiae*; *An. coluzzii*; *An. arabiensis*	Natural	Yes	No	*P. falciparum*: ↓ prevalence	↑ oviposition rate	[11–13]
*w*Anga_Mali	*An. gambiae*; *An. coluzzii*	Natural	Yes	No	*P. falciparum*: ↓ oocysts; ↓ sporozoites	?	[14]
*w*Anga_TZ	*An. arabiensis*	Natural	?	?	?	?	***

In combination with previous evidence from West [11–14], Central and East Africa [15, 16], this confirmation of *Wolbachia* infection in *An. arabiensis* in Tanzania indicates that this endosymbiont may be widespread and ubiquitous in malaria vector populations across the continent. Absence of *Wolbachia* in the 41 *An. funestus* specimens should not be interpreted as absence of the endosymbiont in the species, and that future surveys may find it. This finding should encourage future exploitation of this strain as an agent of malaria control through its potential impact on the transmission capacity of malaria vectors. Further work is crucially needed to understand the ecological, genetic and mechanistic bases of *Wolbachia*-parasite interactions in different *Anopheles* vectors and in different ecological settings. Indeed, this knowledge is required for: (i) the development of this strain as a bio-control agent, similar to ongoing trials for dengue control; (ii) the prediction of how variation of natural *w*Anga infection prevalence influences disease transmission in mosquito populations.

## Conclusions

In the Kilombero Valley (Tanzania), malaria mosquito populations of *An. arabiensis* are naturally infected with *Wolbachia* (*w*Anga_TZ). Understanding its impact on mosquito vectorial capacity is paramount for the development of novel bio-control tools based on this endosymbiont.

## Additional file

Additional file 1: **Figure S1.** Multiple sequence alignment of *16S* rRNA conserved region used for phylogenetic analysis. The consensus sequence is reported together with the consensus and occupancy histograms (using Jalview). Nucleotides are colour coded for clarity. Sequences are ordered based on their pairwise similarity. (TIF 4248 kb)

## Abbreviations

CI: Cytoplasmic incompatibility
EIR: Entomological inoculation rate
GTR +G: General time reversible model
MLST: Multilocus sequence typing
