# Supplementary figures and images for: First report of natural Wolbachia infection in the malaria mosquito Anopheles arabiensis in Tanzania
Source: Parasit Vectors. 2018 Dec 13;11:635. doi: 10.1186/s13071-018-3249-y (PMC6293665; doi:10.1186/s13071-018-3249-y)

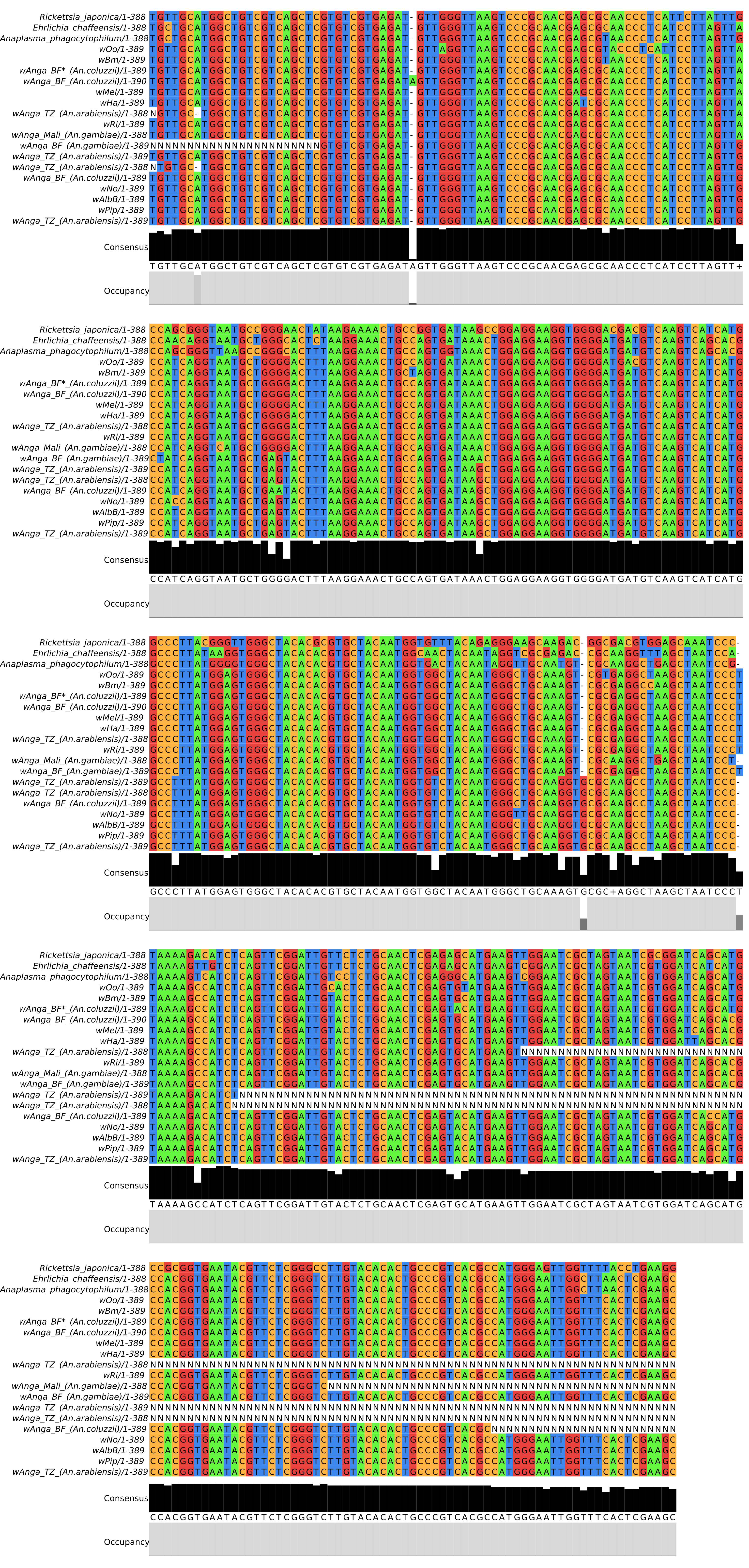

Supplement: Supplementary file 1 — Figure S1. Multiple sequence alignment of 16S rRNA conserved region used for phylogenetic analysis. The consensus sequence is reported together with the consensus and occupancy histograms (using Jalview). Nucleotides are colour coded for clarity. Sequences are ordered based on their pairwise similarity. (TIF 4248 kb) [file 13071_2018_3249_MOESM1_ESM.tif]
